# A Prediction Tool for the Presence of Ceftriaxone-Resistant Uropathogens upon Hospital Admission

**DOI:** 10.3390/antibiotics9060316

**Published:** 2020-06-10

**Authors:** Nancy Yanzhe Li, Gang Quan Poh, Gladys Chung Wei Teng, Hui Hiong Chen, Douglas Su Gin Chan, Siew-Pang Chan, Paul Anantharajah Tambyah, Natasha Bagdasarian, Jia En Wu

**Affiliations:** 1 Department of Pharmacy, National University Health System, Singapore 119228, Singapore; nancyliy@stanford.edu (N.Y.L.); pohgangquan@poissonpharma.sg (G.Q.P.); wei_teng_chung@nuhs.edu.sg (G.C.W.T.); hui_hiong_chen@nuhs.edu.sg (H.H.C.); 2 School of Medicine, Stanford University, Stanford, CA 94305, USA; 3 Department of Laboratory Medicine, National University Health System, Singapore 119228, Singapore; douglas_chan@nuhs.edu.sg; 4 Yong Loo Lin School of Medicine, National University of Singapore, Singapore 117597, Singapore; mdccsp@nus.edu.sg (S.-P.C.); mdcpat@nus.edu.sg (P.A.T.); natasha_bagdasarian@nuhs.edu.sg (N.B.); 5 Cardiovascular Research Institute, National University Heart Centre, Singapore 119074, Singapore; 6 College of Science, Health & Engineering, La Trobe University, Melbourne 8086, Australia; 7 Department of Medicine, National University Health System, Singapore 119228, Singapore

**Keywords:** antibiotic resistance, Asia-Pacific, prediction tool, urinary tract infection, uropathogen

## Abstract

Antimicrobial resistance among uropathogens is a particularly pressing problem in the Asia-Pacific region. The objectives of this study were to determine the incidence and susceptibility of uropathogens upon hospital admission and to develop a risk-scoring model to predict the presence of ceftriaxone-resistance uropathogens (CrP). This was a retrospective observational cohort study of patients with a positive urine culture within 48 h of presentation at National University Hospital, Singapore between June 2015 and August 2015. *Escherichia coli* was the most common uropathogen isolated (51.7%), followed by *Klebsiella pneumonia* (15.1%) and *Pseudomonas aeruginosa* (8.2%). Overall, 372 out of 869 isolates (42.8%) were resistant to ceftriaxone. Hospitalization for ≥2 days within past 30 days, antibiotic use within the past 3 months and male gender were associated with the presence of CrP. A risk score based on these parameters successfully predicted CrP with an area under the curve of 0.68. The risk score will help clinicians to accurately predict antibiotic resistance at the individual patient level and allow physicians to safely prescribe empiric ceftriaxone in patients at low risk of CrP, thus reducing the antibiotic selection pressure that is driving carbapenem resistance in hospitals throughout Asia.

## 1. Introduction

Urinary tract infections (UTIs) are among the most common bacterial infections and a major contributor to global antibiotic use and, consequently, resistance. The choice of empirical antimicrobial therapy should be guided by knowledge of local resistance patterns. However, international guidelines for the treatment of UTIs are largely based on the susceptibility profiles of uropathogens in the United States or European countries [1,2], although several large national surveillance networks for antibiotic-resistant microorganisms have shown that the prevalence of antibiotic resistance in Gram-negative uropathogens varies considerably across the world [2,3].

Ceftriaxone is currently one of the antibiotics recommended for the empiric treatment of UTI in the Asia-Pacific region [4]. However, cephalosporin and fluoroquinolone resistance are on the rise in the Asia-Pacific region [2,5,6]. This has led to concerns about the risks of the widespread use of empiric ceftriaxone therapy.

The objectives of this study were to determine the incidence and susceptibility of uropathogens in a tertiary hospital in Singapore and to develop a risk-scoring model to predict the presence of both ceftriaxone-resistant uropathogens (CrP) and ceftriaxone-resistant Gram-negative uropathogens (CrGNR). This would help clinicians safely use appropriate empiric therapy for patients presenting with UTIs without worsening the antibiotic resistance problem by overly broad-spectrum empiric antibiotic use.

## 2. Results

### 2.1. Study Population and Patient Characteristics

A total of 2063 urinary isolates from 1495 patients between June 2015 and August 2015 were assessed for eligibility. Based on the inclusion and exclusion criteria, a total of 869 isolates from 686 patients were included in this study. A total of 39.4% (270/686) patients were male. While the age range was wide (21–101 years), the majority (73.0%, 501/686) were above 60 years old. Overall, 26.4% (181/686) of the patients had chronic kidney disease, 23.6% (162/686) had instrumentation and 1.7% (12/686) were renal transplant recipients. A total of 42.9% (294/686) of the patients had other forms of urinary tract dysfunctions, mainly comprising ureteric stricture, prostatic hypertrophy, urolithiasis and neurogenic bladder. Among the 686 patients considered, 80.0% (549/686) had one isolate only, 15.5% (106/686) had two isolates only and the remainder had three to five isolates. Out of 869 isolates, 90.6% (787/869) of isolates were Gram-negative bacteria and 42.8% (372/869) of the isolates were CrP. In total, 33.4% (290/869) of the isolates were CrGNR. The sample characteristics are depicted in Table 1.

### 2.2. Distribution of the Types of UTIs and Initial Prescribed Antibiotics for UTI

Majority of the patients had complicated UTI (78.7%, 540/686), out of which one third of these patients had catheter associated UTI as shown in Table 2. Ceftriaxone was the most commonly prescribed antibiotic as empiric therapy for UTI in our institution during the study period (36.6%, 251/686), followed by co-amoxiclav (28.3%, 194/686), piperacillin/tazobactam (15.5%, 106/686) and ciprofloxacin (7.0%, 48/686).

### 2.3. Species Distribution of Uropathogens and Susceptibility of Uropathogens to Common Antibiotics

**Figure 1:**
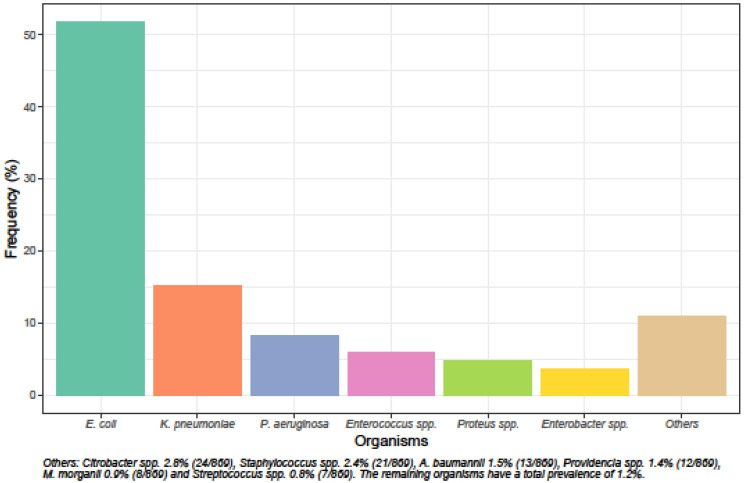
Species distribution of uropathogens.

**Figure 2:**
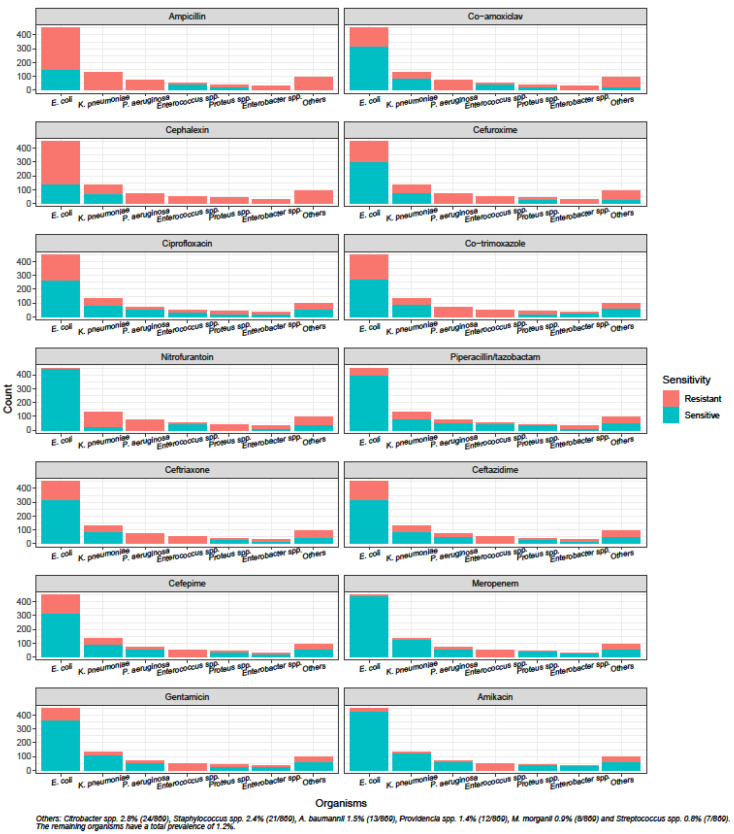
Susceptibility of uropathogens to antibiotics.

The species distribution and susceptibility profiles of different microorganisms to commonly prescribed oral and intravenous antibiotics are shown in Figure 1 and Figure 2. *Escherichia coli* was the most common uropathogen isolated (51.7%, 449/869), followed by *Klebsiella pneumoniae* (15.1%, 131/869), *Pseudomonas aeruginosa* (8.2%, 71/869), *Enterococcus* spp. (5.9%, 51/869), *Proteus* spp. (4.7%, 41/869) and *Enterobacter* spp. (3.6%, 31/869).

The susceptibilities of uropathogens towards commonly prescribed oral antibiotics such as co-amoxiclav, ciprofloxacin and co-trimoxazole were below 70%. Although susceptibility to ceftriaxone was low at 57.2% (497/869), the susceptibilities to piperacillin/tazobactam and gentamicin were higher at 78.8% (685/869) and 74.2% (645/869), respectively. Carbapenems and amikacin were the only antibiotics to demonstrate susceptibilities above 80%.

### 2.4. Risk Factors for the Presence of CrP and CrGNR

The exploratory CHAID algorithm and confirmatory analysis with multilevel gSEM identified antibiotic use within the past 3 months (*p*<0.001), hospitalization for ≥2 days within the past 30 days (*p*<0.001) and male gender (*p*<0.001) as important and statistically significant predictors for the presence of CrP (Table 3). A patient with UTI who had been hospitalized for ≥2 days within the past 30 days had a higher likelihood of CrP UTI, with the odds tripled. A similar risk profile was observed for patients who had antibiotic use within the past 3 months, as their odds were increased by almost 2.6 times. Other things being equal, male patients were more likely to develop CrP UTIs (AOR: 2.66) when compared with their female counterparts.

The effects of catheterization and gender were considered in the model for analyzing CrGNR (Table 3), although none of the candidate predictors were significant. The predictors were retained in view of the magnitudes of the AORs. The odds of developing CrGNR were raised by 58% with catheterization.

The risk-scoring models possessed over 95% statistical power in view of their parsimonious nature, random-intercept structure and sample size.

### 2.5. External Validity of the Risk-Scoring Model

**Figure 3:**
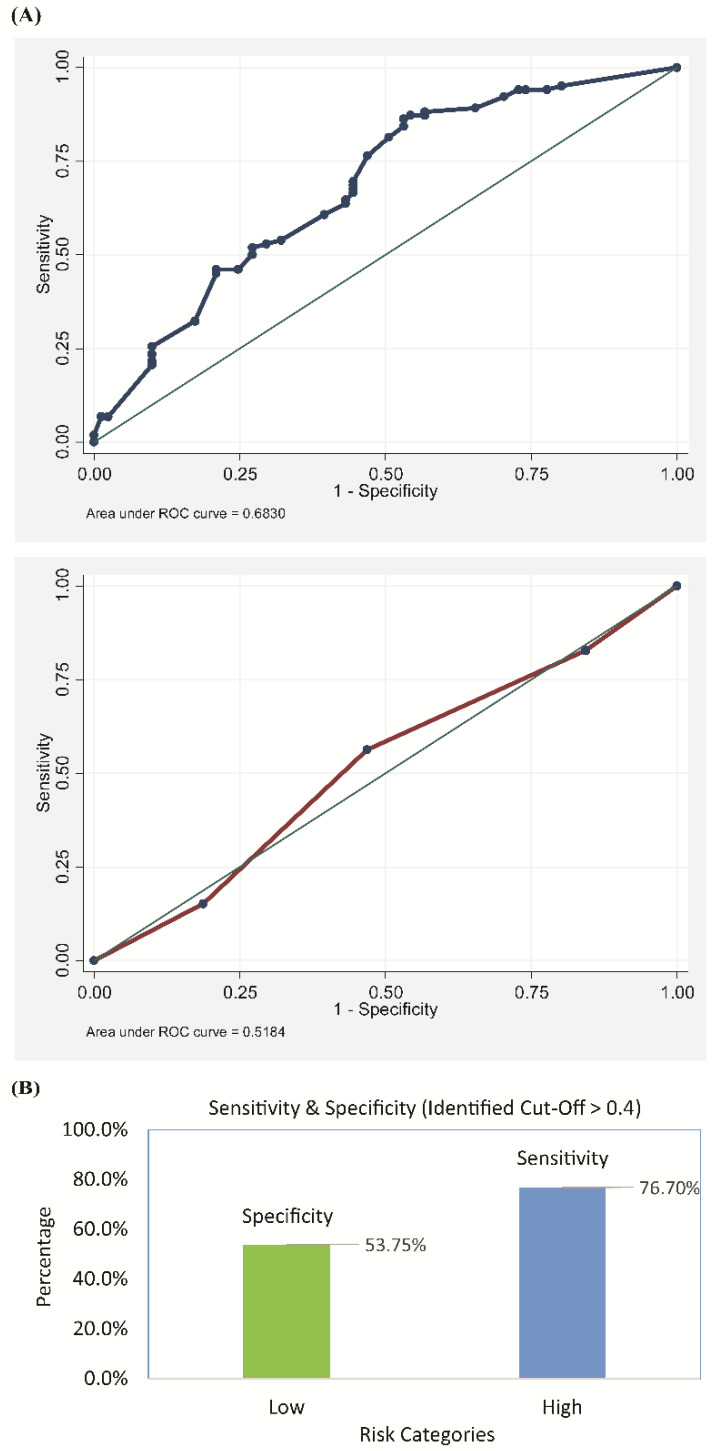
Performance of the risk-scoring model in predicting the presence of ceftriaxone-resistant uropathogens as determined by (**A**) the receiver operating characteristic curve (ROC) and (**B**) the sensitivity and specificity score of the model.

Based on the test subsample of 183 records, the risk-scoring model was found to be adequate in predicting the presence of CrP. As depicted in Figure 3, the area under the ROC curve was 0.68 (95% C.I.: 0.60–0.76). The auxiliary model, based on the training subsample of 686 records and the same predictors, generated similar odds ratios as the full model reported in Table 3. It also provided a satisfactory fit to the data (Hosmer–Lemeshow *p*: 0.30).

The generated probabilities for the presence of CrP could be optimally discretized at 0.40 (sensitivity: 76%, specificity: 53%, accuracy: 66.1%). That is, patients with UTI and a probability of 0.40 and above (based on the three identified predictors) were considered to be at risk for CrP UTI.

## 3. Discussion

*E. coli*, *K. pneumonia* and *P. aeruginosa* were the three most common causative microorganisms for UTI in our population, consistent with results from the Study for Monitoring Antimicrobial Resistance Trends (SMART) from the region conducted in 2013 [6]. Findings from our study have important implications for the initial antibiotic choice for UTI. One third of the *E. coli* and *K. pneumoniae* isolates were resistant to ceftriaxone, and all *Pseudomonas* spp. are intrinsically resistant to ceftriaxone; however, ceftriaxone is among the most widely used antibiotics for the empiric treatment of UTI in our institution.

The Infectious Diseases Society of America suggest a threshold of 20% as the resistance prevalence at which the agent is no longer recommended for empirical treatment of acute cystitis; this may no longer be feasible in the Asia-Pacific region, where Gram-negative resistance is escalating rapidly [1,5,6]. In fact, our study showed that carbapenems and amikacin were the only antibiotics with susceptibilities above 80%. However, the need to conserve the use of broad-spectrum antibiotics such as carbapenems to combat the rise of even worse antimicrobial resistance and the high rates of uropathogen resistance to ceftriaxone highlight the need to accurately predict antibiotic resistance at the individual patient level in our setting, in order to enable better selection of initial antibiotic treatment which may lead to better outcomes, while possibly reducing unnecessary broad-spectrum antibiotic use.

This study found hospitalization for ≥2 days within the past 30 days, antibiotic use within the past 3 months and male gender to be associated with the presence of CrP. With this knowledge, physicians may be able to still safely prescribe empiric ceftriaxone in patients at low risk for CrP and consider aminoglycosides in those with high risk of CrP, thus reserving piperacillin/tazobactam or carbapenems for severely ill patients at high risk of CrP. This should be validated in a large prospective study in other regional sites. This study is also limited by the availability of antibiotics in the institution, and susceptibilities to flomoxef or cefmetazole were not investigated. Further studies are also required to (1) investigate patient outcomes when utilizing the risk score assessment and (2) investigate the risk factors for other antibiotics, such as fluoroquinolones. Other healthcare facilities may have different risk factors and antimicrobial resistance profiles. We think that this approach might be useful for others to consider so that a generalizable approach that could work broadly in the Asia Pacific region might emerge. While there is a need for early appropriate antibiotic therapy, the collateral damage can be considerable. Hopefully, narrowing empiric therapy for community-onset UTIs will reduce the antibiotic pressure that is driving carbapenem resistance in hospitals throughout Asia.

## 4. Materials and Methods

### 4.1. Patient Population, Study Design and Data

The National University Hospital (NUH) is a tertiary care hospital in Singapore with 1250 inpatient beds. Patients with a positive urine culture within 48 h of presentation at NUH between June 2015 and August 2015 were included in this retrospective observational cohort study. The exclusion criteria were (a) age below 21 years, (b) admission to intensive care unit and (c) those with asymptomatic bacteriuria that were not in a high-risk group for whom therapy is indicated (pregnancy or undergoing urological procedures). The study was approved by the Domain-Specific Review Board ethics committee (reference number: 2015/00806) and adhered to the Strengthening the Reporting of Observational Studies in Epidemiology guidelines for reporting [7].

The potential risk factors for presence of resistant uropathogens were identified based on the existing literature. These included advanced age, male gender, institutionalization, urinary catheterization, diabetes, renal transplant, immunodeficiency, chronic kidney disease, structural/functional abnormality of the urinary tract, prior urological procedures, antibiotic use within the past 3 months, positive urine culture within the past 3 months, hospitalization for ≥2 days within the past 3 months or within 30 days, having received home wound care, chemotherapy or hemodialysis within the past 30 days [8,9,10].

All relevant patient data (i.e., demographics, past medical history, diagnosis, antibiotic prescription) were extracted from the electronic medical records. Microbiological information was obtained from the Department of Laboratory Medicine. Antimicrobial susceptibility testing was performed in the NUH College of American Pathologists (CAP) accredited laboratory, predominantly through the Vitek 2 system (bioMe′rieux, Marcy l’Etoile, France), and interpreted according to European Committee for Antimicrobial Susceptibility Testing (EUCAST) standards.

### 4.2. Data Analysis

Quantitative data were expressed as mean ± standard deviation, while qualitative data were presented in counts and percentages. The chi-square automated interactions detector (CHAID) [11], a multiway decision tree model, was applied for preliminary identification of important risk factors associated with the presence of CrP and CrGNR. This was achieved by computation of the importance index, which documented the reduction in predictive accuracy should the risk factors be omitted. CHAID, owing to its multiway splitting algorithm, enabled identification of the optimal cut-off(s) of the quantitative risk factors. The associations between the selected risk factors and the outcomes were estimated with a logit model within the multilevel generalized structural equation modeling (gSEM) framework [12], with further model refinement carried out with a backward elimination procedure (removal probability >0.05). However, the magnitudes of the adjusted odds ratios were taken into account when selecting the models. The multilevel technique was considered in anticipation that individual patients with multiple episodes would be reported. The standard errors of the estimated adjusted odds ratios (AOR) were refined with a robust procedure. The training subsample comprised the first episode for each patient, while the subsequent episodes made up the test subsample where the risk-scoring model’s predictive accuracy based on the receiver operating characteristic curve (ROC) was ascertained. Analyzed with Stata MP v14 (Stata Corp. College Station, TX, USA), all statistical tests were performed at a 5% level of significance.

## Figures and Tables

**Table 1:** Sample Characteristics.

Variables	
Patient-Level Data	*n* = 686
Age in years (mean ± SD)	69.0 ± 18.7
Race, *n* (%)	
Chinese	443 (64.6)
Malay	136 (19.8)
Indian	74 (10.8)
Others	33 (4.8)
Gender, *n* (%)	
Female	416 (60.6)
Male	270 (39.4)
Diabetes mellitus, *n* (%)	276 (40.2)
Chronic Kidney Disease, *n* (%)	181 (26.4)
Renal transplant, *n* (%)	12 (1.7)
Hemodialysis within the past 30 days, *n* (%)	8 (1.2)
Immunodeficiency, *n* (%)	44 (6.4)
Prior urological procedure, *n* (%)	54 (7.9)
Antibiotic use within the past 3 months, *n* (%)	231 (33.7)
Positive urine culture within the past 3 months, *n* (%)	157 (22.9)
Structural/functional abnormality of urinary tract, *n* (%)	294 (42.9)
Received chemotherapy infusion within past 30 days, *n* (%)	8 (1.2)
Home wound care within past 30 days, *n* (%)	45 (6.6)
Hospitalization for ≥2 days within the past 3 months, *n* (%)	233 (34.0)
Hospitalization for ≥2 days within the past 30 days, *n* (%)	149 (21.7)
Catheterization, *n* (%)	162 (23.6)
Intermittent	21 (3.1)
Suprapubic	4 (0.6)
Indwelling	137 (20.0)
Duration of catheterization, *n* (%)	
Short term (<30 days)	24 (3.5)
Long term (≥30 days)	138 (20.1)

**Table 2:** Types of UTIs.

Diagnosis	Prevalence (%)
Uncomplicated UTI	132 (19.2)
Cystitis	54 (7.9)
Pyelonephritis/Pyonephrosis ^	78 (11.4)
Complicated UTI *	540 (78.7)
Pyelonephritis/Pyonephrosis ^	157 (22.9)
Prostatitis/Epididymo-orchitis	5 (0.7)
Catheter associated UTI	161 (23.5)
ASB (pregnancy)	10 (1.5)
ASB (planning for invasive urological procedures)	4 (0.6)

* UTI with presence of underlying structural or functional abnormalities of the urinary tract, catheterization, renal diseases, other concomitant immunocompromising diseases and/or male UTI. ^ UTI with symptoms of fever, nausea, vomiting, frank pain, costovertebral angle tenderness and/or altered mental status.

**Table 3:** Analysis of the presence of ceftriaxone-resistant uropathogens and ceftriaxone-resistant Gram-negative uropathogens (GNR) with multilevel generalized structural equation modeling.

Risk Factor	Ceftriaxone-Resistant AOR (95% C.I.)	*p*-Value	Ceftriaxone-Resistant GNR AOR (95% C.I.)	*p*-Value
Hospitalization for ≥2 days within past 30 days	3.05 (1.79 − 5.22)	<0.001	NA	NA
Antibiotic use within the past 3 months	2.56 (1.60 − 4.08)	<0.001	NA	NA
Male gender	2.66 (1.66 − 4.25)	<0.001	0.67 (0.41 − 1.11)	0.124
Catheterization	NA	NA	1.58 (0.90 − 2.77)	0.110

## Abbreviations

UTI	Urinary tract infection
ASB	asymptomatic bacteriuria
CrP	ceftriaxone-resistant uropathogens
CrGNR	ceftriaxone-resistant Gram-negative uropathogens
NUH	National University Hospital
CAP	College of American Pathologists
EUCAST	European Committee for Antimicrobial Susceptibility Testing
CHAID	chi-square automated interactions detector
gSEM	generalized structural equation modeling
AOR	adjusted odds ratios
ROC	receiver operating characteristic
SMART	Study for Monitoring Antimicrobial Resistance Trends
SD	standard deviation
C.I.	confidence interval
GNR	Gram-negative uropathogens
